# Machine-learning-derived radiomics signature of pericoronary tissue in coronary CT angiography associates with functional ischemia

**DOI:** 10.3389/fphys.2022.980996

**Published:** 2022-09-26

**Authors:** Yan Feng, Zhihan Xu, Lin Zhang, Yaping Zhang, Hao Xu, Xiaozhong Zhuang, Hao Zhang, Xueqian Xie

**Affiliations:** ^1^ Department of Radiology, Shanghai General Hospital of Nanjing Medical University, Shanghai, China; ^2^ Department of Radiology, Shanghai General Hospital, Shanghai Jiao Tong University School of Medicine, Shanghai, China; ^3^ DI CT Collaboration, Siemens Healthineers Ltd, Shanghai, China; ^4^ Department of Cardiology, Shanghai General Hospital, Shanghai Jiao Tong University School of Medicine, Shanghai, China

**Keywords:** machine learning, fractional flow reserve, radiomics, functional ischemia, coronary computed tomography angiography

## Abstract

**Objectives:** To determine the association between radiomics signature (Rad-signature) of pericoronary tissue (PCT) in coronary computed tomography angiography (CCTA) and CT-derived fractional flow reserve (CT-FFR), and explore the influential factors of functional ischemia.

**Methods:** We retrospectively included 350 patients who underwent CCTA from 2 centers, consisting of the training (*n* = 134), validation (*n* = 66), and testing (with CCTA and invasive coronary angiography, *n* = 150) groups. After evaluating coronary stenosis level in CCTA (anatomical CT), pericoronary fat attenuation index (FAI), and CT-FFR, we extracted 1,691 radiomic features from PCT. By accumulating and weighting the most contributive features to functional ischemia (CT-FFR ≤ 0.8) the Rad-signature was established using Boruta integrating with a random forest algorithm. Another 45 patients who underwent CCTA and invasive FFR were included to assure the performance of Rad-signature.

**Results:** A total of 1046 vessels in 350 patients were analyzed, and functional ischemia was identified in 241/1046 (23.0%) vessels and 179/350 (51.1%) patients. From the 47 features highly relevant to functional ischemia, the top-8 contributive features were selected to establish Rad-signature. At the vessel level, the area under the curve (AUC) of Rad-signature to discriminate functional ischemia was 0.83, 0.82, and 0.82 in the training, validation, and testing groups, higher than 0.55, 0.55, and 0.52 of FAI (*p* < 0.001), respectively, and was higher than 0.72 of anatomical CT in the testing group (*p* = 0.017). The AUC of the combined model (Rad-signature + anatomical CT) was 0.86, 0.85, and 0.83, respectively, significantly higher than that of anatomical CT and FAI (*p* < 0.05). In the CCTA-invasive FFR group, using invasive FFR as the standard, the mean AUC of Rad-signature was 0.83 ± 0.02. At the patient level, multivariate logistic regression analysis showed that Rad-signature of left anterior descending (LAD) [odds ratio (OR) = 1.72; *p* = 0.012] and anatomical CT (OR = 3.53; *p* < 0.001) were independent influential factors of functional ischemia (*p* < 0.05). In the subgroup of nonobstructive (stenosis <50% in invasive coronary angiography) and obstructive (≥50%) cases of the testing group, the independent factor of functional ischemia was FAI of LAD (OR = 1.10; *p* = 0.041) and Rad-signature of LAD (OR = 2.45; *p* = 0.042), respectively.

**Conclusion:** The machine-learning-derived Rad-signature of PCT in CCTA demonstrates significant association with functional ischemia.

## Introduction

Functional ischemia is a state in which the blood flow cannot meet the metabolic needs of tissues even in the absence of vascular obstruction (Moroni et al., 2021). Coronary computed tomography angiography (CCTA) is widely used for demonstrating the degree of coronary stenosis, but lacks the information of blood flow function. Invasive fractional flow reserve (FFR) is the gold standard for assessing coronary blood flow, and used for clinical decision-making in the treatment of coronary artery disease (CAD) (Pijls et al., 2007; Tonino et al., 2009; De Bruyne et al., 2012; Windecker et al., 2014). However, its clinical application is limited due to the invasive and high-cost pressure guide wire. Recently, CT-derived FFR (CT-FFR) based on hydrodynamics or deep learning has been developed to noninvasively measure lumen blood flow, avoiding additional radiation exposure and invasive procedure. A CT-FFR value ≤0.8 is considered coronary functional ischemia, and studies have proved that CT-FFR is highly consistent with invasive FFR (Tesche et al., 2017; Alex et al., 2020). In addition to assessing blood flow from the perspective of lumen, exploring the association between functional ischemia and pericoronary tissue (PCT) may provide more evidence for the diagnosis and treatment of CAD.

The bidirectional interaction between pericoronary adipose tissue (PCAT) and the adjacent coronary wall leads to coronary artery inflammation and plaque formation (Margaritis et al., 2013; Antonopoulos et al., 2014; Antonopoulos et al., 2015; Antonopoulos et al., 2017). Then, inflammatory cell infiltration and edema in PCAT result in increased CT attenuation, realizing the visualization and quantitative evaluation of vascular inflammation. A PCAT imaging biomarker, fat attenuation index (FAI), has been introduced as a strong and independent predictor of major adverse cardiovascular events (Dai et al., 2022). Ma et al. (2021) found that overall FAI was not significantly associated with abnormal FFR, but lesion-specific PCAT was independently related to abnormal FFR. FAI only incorporates PCAT density, but does not reflect the complex tissue structures around the coronary artery. Although the widely used CT attenuation range of PCAT is from −190 to −30 Hounsfield unit (HU), the PCAT attenuation of high-risk plaque with a “fat stranding” sign can reach 31 HU, because of the complex plaque components (Hedgire et al., 2018). In order to analyze PCT, machine learning-based radiomics allows to extract and analyze numerous quantitative features inside the medical images (Gillies et al., 2016).

Therefore, we hypothesize that the radiomic features of PCT are associated with functional coronary ischemia. Considering that some information may be omitted when solely determining the adipose tissue by CT attenuation, we aim to comprehensively analyze PCT by extracting the radiomic features of adipose and other tissues around the coronary artery. We established a machine-leaning-derived radiomics signature (Rad-signature) based on PCT to discriminate functional ischemia, compared with the conventional stenosis grading on CCTA (anatomical CT) and FAI, and then analyzed the influential factors of functional ischemia.

## Materials and methods

### Study sample

The patients were retrospectively included in two medical centers [Hospital-1: Shanghai General Hospital-North (city center); Hospital-2: Shanghai General Hospital-South (Songjiang new city)]. The inclusion criteria of subjects with CCTA were as follows: 1) patients with suspected or diagnosed CAD, defined by the guidelines (Fox et al., 2006; Fihn et al., 2012; Montalescot et al., 2013); 2) patients with CCTA from January to December 2020 to establish the model and validate its performance; 3) patients who underwent CCTA and invasive coronary angiography (ICA) from January 2014 to December 2019 to test the model and analyze the influential factors of functional ischemia by subgrouping the patients into obstructive and nonobstructive CAD. Additionally, patients who underwent CCTA and invasive FFR from January 2020 to December 2021 were included to assure the performance of Rad-signature in discriminating standard functional ischemia.

The exclusion criteria were: 1) history of coronary stenting or bypass surgery; 2) poor image quality and insufficient for diagnosis; 3) coronary artery variation; 4) total occlusion of coronary artery limiting the calculation of CT-FFR; 5) images that cannot be processed by the post-processing workstation, resulting in the failure of CT-FFR calculation or PCT segmentation; 6) time interval between CCTA and ICA (or invasive FFR) >2 months.

**FIGURE 1:**
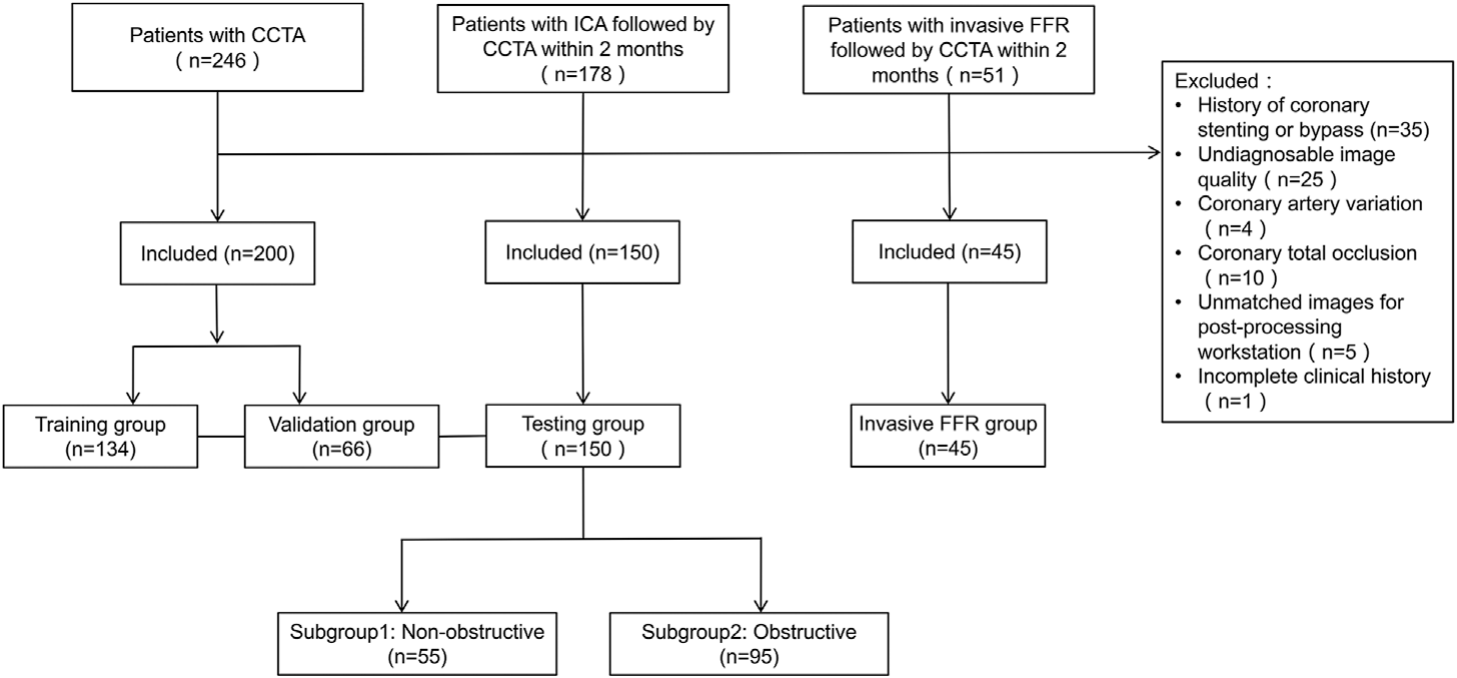
Flowchart of patient selection and grouping. CCTA, coronary computed tomography angiography; CAD, coronary artery disease; ICA, invasive coronary angiography.

The patients were divided into four groups, including the training, validation (patients with CCTA), testing (with CCTA and ICA), and CCTA-invasive FFR groups. Figure 1 displays patient selection and grouping. The institutional review board approved this retrospective study and exempted the patient informed consent.

In the training, validation, and testing groups, a CT-FFR value ≤0.8 was defined as coronary functional ischemia (Tesche et al., 2017; Alex et al., 2020). In the invasive FFR group, an invasive FFR value ≤ 0.8 was defined as standard coronary functional ischemia. Basic characteristics and medical history were collected from the electronic medical record system, including age, sex, body mass index (BMI), smoking history, family history of CAD, diabetes, hypertension, and hyperlipidemia.

### Coronary computed tomography angiography acquisition and anatomic evaluation

CCTA examination was performed on different CT equipment (Somatom Definition Flash and Somatom Force, Siemens Healthineers; Revolution CT and Discovery750 HD, GE Healthcare; Aquilion ONE, Canon Medical Systems) in the two centers (Supplementary Table S1). Beta-blockade was administrated for patients with a heart rate >90 beats per minute. Prospective gated CT scanning was performed. The scan covering range was from 1 cm below the tracheal carina to 2 cm below the left diaphragm. The tube voltage and current were automatically set using smart mode. The volume of contrast medium (Iopamidol 370 mg I/mL; Bracco) was calculated as 0.8 ml/kg, and the flow rate was 4–5 ml/sec. Then 20 ml of normal saline was injected at the same flow rate.

According to the standard segments of coronary artery recommended by the Society of Cardiovascular Computed Tomography of America (Leipsic et al., 2014), two experienced radiologists with 5- and 15-years experience in cardiovascular imaging independently evaluated the diameter stenosis level of the left anterior descending (LAD), left circumflex (LCx), and right coronary artery (RCA), using a dedicated image processing workstation (Advanced Workstation 4.6, GE Healthcare) with curved planar reformation, multiplanar reformation and volume rendering, and resolved the disagreement by mutual consultation. The vessels of <1.5 mm in diameter were excluded from anatomical evaluation.

### Invasive coronary angiography and invasive fractional flow reserve methods

Invasive coronary angiography was performed by experienced interventional cardiologists according to local clinic standards, who were blinded to the results of CCTA. After radial artery or femoral artery puncture, left and right coronary angiography was performed. Based on the 5 projection angles of the left coronary artery and 2 of the right coronary artery, the degree of stenosis was quantitatively determined. A stenosis level ≥50% was considered obstructive CAD. An interventional cardiologist performed invasive FFR measurements based on coronary stenosis to determine the hemodynamic significance and the need for revascularization. The FFR pressure wire (Pressure Wire Abbott, St. Jude Medical, United States) was placed distal to the stenosis position. An FFR ≤ 0.80 was considered functional ischemia. The dose of contrast agent for each view was approximately 5 ml. The X-ray dose pattern was electrophysiological. The image acquisition rate was 7.5 frames per second.

### Functional CT-derived fractional flow reserve

An on-site research prototype application (cFFR v3.5.0, Siemens Healthineers, not currently commercially available) was implemented for CT-FFR computation, which has been previously described and validated (Itu et al., 2016; Coenen et al., 2018; Tesche et al., 2018). This application calculates the CT-FFR value using a deep learning-based framework, which integrates the complex nonlinear relationship between the various features extracted from the coronary tree geometry and computes the blood flow of a coronary position (Itu et al., 2016). This model calculates CT-FFR based on reduced-order hydrodynamics. This application program can segment the coronary artery lumen on CT images to generate a coronary tree, and semiautomatically represent the CT-FFR value of any point in this tree. Before calculating CT-FFR, the observer needs to confirm or manually edit the automatically recognized coronary centerlines, and then the software generates a 3D pseudocolor map to comprehensively visualize the CT-FFR values of the coronary artery tree.

A radiologist with 10 years of cardiac imaging experience measured CT-FFR values, blinded to the results of medical history and other examinations. In this study, the CT-FFR values of LAD, LCx, and RCA were recorded. For vessels with stenosis, the measuring position was 2–3 cm distal from the stenosis. In the case of multiple stenoses in a single vessel, the distal end of the farthest lesion was measured. For normal vessels, the measuring position was the farthest end (about 1.5 mm in diameter). In order to match the positions of the invasive FFR and CT-FFR measurements, an independent radiologist, blinded to the functional results, marked the corresponding location on the CT-FFR image after identifying the location of the invasive FFR on the fluoroscopic image.

### Pericoronary tissue segmentation and radiomic feature extraction

The same radiologist segmented PCT using dedicated software (Coronary plaque analysis v5.0.2, Frontier, Syngo. *via*, Siemens Healthineers). The software automatically segmented the image, and the radiologist manually modified them in case of inaccuracy. PCT is defined as all voxels extending outward from the outer wall of the vessel with a radius equal to the vessel diameter (Goeller et al., 2019). For LAD and LCx, the analyzed PCT was 4 cm long in the proximal segment of the vessels. For RCA, the analyzed tissue was 4 cm long in the proximal segment (1–5 cm from RCA ostium). The software then calculated FAI based on the segmented volume.

After importing the PCT mask, the dedicated software (Radiomics 13.0, Frontier, Syngo. *via*, Siemens Healthineers) automatically extracted and calculated 1,691 radiomic features of each vessel in about 10 s, including three major categories: 18 first-order, 75 texture, and 17 size and shape features.

### Feature selection and rad-signature construction

**FIGURE 2:**
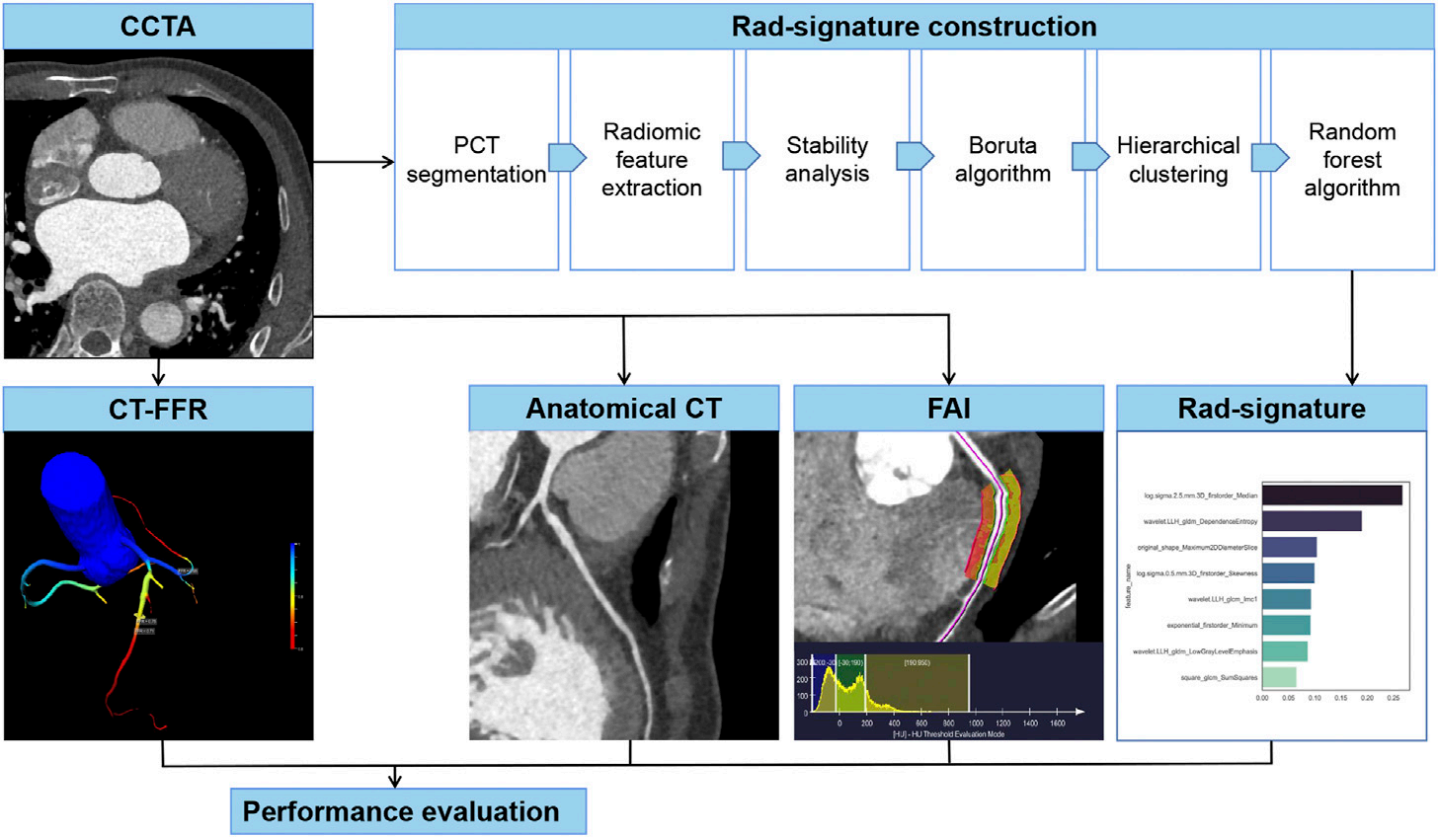
Establishment and evaluation of anatomical CT, FAI, and Rad-signature. CCTA, coronary computed tomography angiography. PCT, pericoronary tissue. CT-FFR, CT-derived fraction flow reserve; FAI, fat attenuation index.

To select stable and repeatable features, a radiologist with 10 years of experience in cardiac imaging randomly selected 30 vessels from the training group, segmented PCT and extracted radiomic features, and repeated the same procedure 1 month later. In these two measurements, the features with an intraclass correlation coefficient >0.8 were considered stable. Boruta algorithm integrated with random forest selected highly associated features by iteratively deleting features. Then the Boruta algorithm-selected features were converged by hierarchical clustering, and the most important features were selected as candidate features from each cluster. In this study, a random forest algorithm constructed a Rad-signature incorporating multiple features into one value (Dercle et al., 2020; Wu et al., 2020). The parameters in the training group were estimated by grid search with 10-fold cross-validation to avoid overfitting. The feature importance was assessed by the Gini impurity decreased overall decision trees. Each coronary vessel has a Rad-signature with a rad-score range of 0–1, indicating the probability of functional ischemia. The greater the Rad-signature, the more likely functional ischemia happens. In the CCTA-invasive FFR group, 4-fold cross-validation was used to estimate the performance of Rad-signature in discriminating standard functional ischemia. Figure 2 shows the process of establishing Rad-signature and performance evaluation. Supplementary Figure S1 demonstrates a representative case of Rad-signature establishment.

### Statistics

Continuous variables were represented by median (25% and 75% quartile), and the difference between groups was tested by the Mann-Whitney *U* test. Categorical variables were expressed by frequency (percentages) and tested by Chi-Square test. At the vessel level, the performance of Rad-signature, anatomical CT, and FAI in identifying functional ischemia (CT-FFR ≤ 0.8) was evaluated by receiver operating characteristic (ROC) curve, area under the curve (AUC), accuracy, sensitivity, and specificity. The optimal cut-off value was indicated by Youden’s index based on the training group. The difference between AUCs was evaluated by Delong’s test. The incremental value of Rad-signature to anatomical CT was further evaluated by net reclassification index. At the patient level, the vessel with the lowest CT-FFR was used to define functional ischemia. The potential risk factors of functional ischemia were explored in the entire study sample by univariate and multivariate logistic regression analysis. Additionally, in the testing group (with CCTA and ICA), subgroup analysis was implemented to further assess the correlation between Rad-signature and functional ischemia in different risk levels (obstructive or nonobstructive CAD). The statistics was performed by open-source packages (R v3.6.0, http://www.Rproject.org; Python v3.7 with Scikit-survival library v0.13.2, https://scikit-survival.readthedocs.io/en/latest/). Supplementary Table S2 lists the details of software packages and functions. A *p* < 0.05 was considered statistically significant.

## Results

### Patient and vessel characteristics

In the training, validation, and testing groups, a total of 350 patients (66 years; 61–71 years) were eligible for this study from 442 candidates, including 134, 66, and 150 in the training, validation, and testing groups, respectively. In the three groups, 1046 vessels were analyzed including 350 LAD, 346 LCx, and 350 RCA, and functional ischemia (CT-FFR ≤ 0.8) was identified in 241/1046 (23.0%) vessels and 179/350 (51.1%) patients. In the three groups, anatomical CT detected coronary stenosis ≥50% in 377/1046 (36.0%) vessels and 236/350 (67.4%) patients. At the vessel level, the median FAI was −83.5 HU (−89.7 to −77.6 HU). In the testing group, ICA diagnosed obstructive CAD (stenosis ≥50%) in 148/447 (33.1%) vessels and in 95/150 (63.0%) patients. Tables 1, 2 summarize the clinical and CT characteristics of the three groups, respectively.

**TABLE 1:** Clinical characteristics.

Characteristics	All	Training group	Validation group	Testing group	*p*-Value
	*n* = 350	*n* = 134	*n* = 66	*n* = 150	
Male	208 (59.4%)	79 (59.0%)	41 (62.1%)	88 (58.7%)	0.884
Age, years	66.0 [61.0; 71.0]	66.5 [62.0; 71.0]	65.0 [59.5; 71.0]	65.0 [60.0; 71.0]	0.599
Hypertension	197 (56.3%)	76 (56.7%)	37 (56.1%)	84 (56.0%)	0.992
Diabetes	124 (35.4%)	64 (47.8%)	23 (34.8%)	37 (24.7%)	**<0.001**
Hyperlipemia	124 (35.4%)	51 (38.1%)	28 (42.4%)	45 (30.0%)	0.153
Smoking	94 (26.9%)	23 (17.2%)	23 (34.8%)	48 (32.0%)	**0.005**
Family history of CAD	29 (8.29%)	10 (7.46%)	11 (16.7%)	8 (5.33%)	**0.019**
Body mass index, kg/m^2^	24.3 [22.0; 26.4]	24.2 [21.5; 26.3]	24.3 [22.0; 26.8]	24.2 [22.4; 26.4]	0.678

Data are represented by median [25% and 75% quartile] or frequency (percentage).

*p*-Value represents the difference among the train, validation, and testing groups.

Bold values signify statistical significance.

CAD, coronary artery disease.

**TABLE 2:** CT characteristics.

Variables	Training group	Validation group	Testing group	*p*-value
	*n* = 134	*n* = 66	*n* = 150	
LVM-CT	127 [108; 151]	138 [113; 163]	134 [115; 152]	0.221
CT-FFR_RCA	89.0 [84.0; 93.0]	91.0 [86.0; 94.0]	92.0 [86.0; 94.0]	**0.011**
≤0.8	26 (19.4%)	8 (12.1%)	13 (8.7%)	
>0.8	108 (80.6%)	58 (87.9%)	137 (91.3%)	
CT-FFR_LAD	80.5 [67.0; 86.0]	80.5 [66.2; 87.8]	85.0 [76.0; 90.0]	**0.001**
≤0.8	67 (50%)	33 (50%)	52 (34.7%)	
>0.8	67 (50%)	33 (50%)	98 (65.3%)	
CT-FFR_LCx	91.0 [85.0; 94.8]	92.0 [87.0; 95.0]	92.0 [86.0; 95.0]	0.816
≤0.8	18 (13.4%)	7 (10.8%)	17 (11.6%)	
>0.8	116 (86.6%)	58 (89.2%)	130 (88.4%)	
CT-FFR_patient	76.0 [63.5; 84.0]	77.5 [65.0; 85.0]	82.5 [71.0; 88.0]	**0.001**
≤0.8	78 (58.2%)	37 (56.1%)	64 (42.7%)	
>0.8	56 (41.8%)	29 (43.9%)	86 (57.3%)	
FAI_LAD	−87.18 [−92.52; −82.17]	−84.08 [−92.75; −78.84]	−84.97 [−91.15; −78.18]	0.082
FAI_RCA	−86.29 [−91.90; −79.47]	−84.04 [−91.46; −79.78]	−84.16 [−90.22; −78.21]	0.229
FAI_LCx	−79.86 [−85.44; −75.03]	−79.45 [−83.70; −74.01]	−79.83 [−87.09; −74.06]	0.745
Anatomical CT_RCA				0.138
<50%	86 (64.2%)	48 (72.7%)	112 (74.7%)	
≥50%	48 (35.8%)	18 (27.3%)	38 (25.3%)	
Anatomical CT_LAD				0.125
<50%	50 (37.3%)	29 (43.9%)	74 (49.3%)	
≥50%	84 (62.7%)	37 (56.1%)	76 (50.7%)	
Anatomical CT_LCx				0.991
<50%	105 (78.4%)	52 (78.8%)	117 (78.0%)	
≥50%	29 (21.6%)	14 (21.2%)	33 (22.0%)	
Anatomical CT_patient				0.404
<50%	38 (28.4%)	24 (36.4%)	52 (34.7%)	
≥50%	96 (71.6%)	42 (63.6%)	98 (65.3%)	

LVM-CT, left ventricular mass on CT; CT-FFR, CT-derived fraction flow reserve; FAI, fat attenuation index.

Data are represented by median [25% and 75% quartile] or frequency (percentage).

Bold values signify statistical significance.

The CCTA-invasive FFR group included 55 vessels in another 45 patients (65 years; 59–71 years), including 7 RCA, 37 LAD, and 11 LCx. Functional ischemia (FFR ≤ 0.8) was detected in 13/55 (23.6%) vessels and 11/45 (24.4%) patients.

### Radiomics feature selection

**FIGURE 3:**
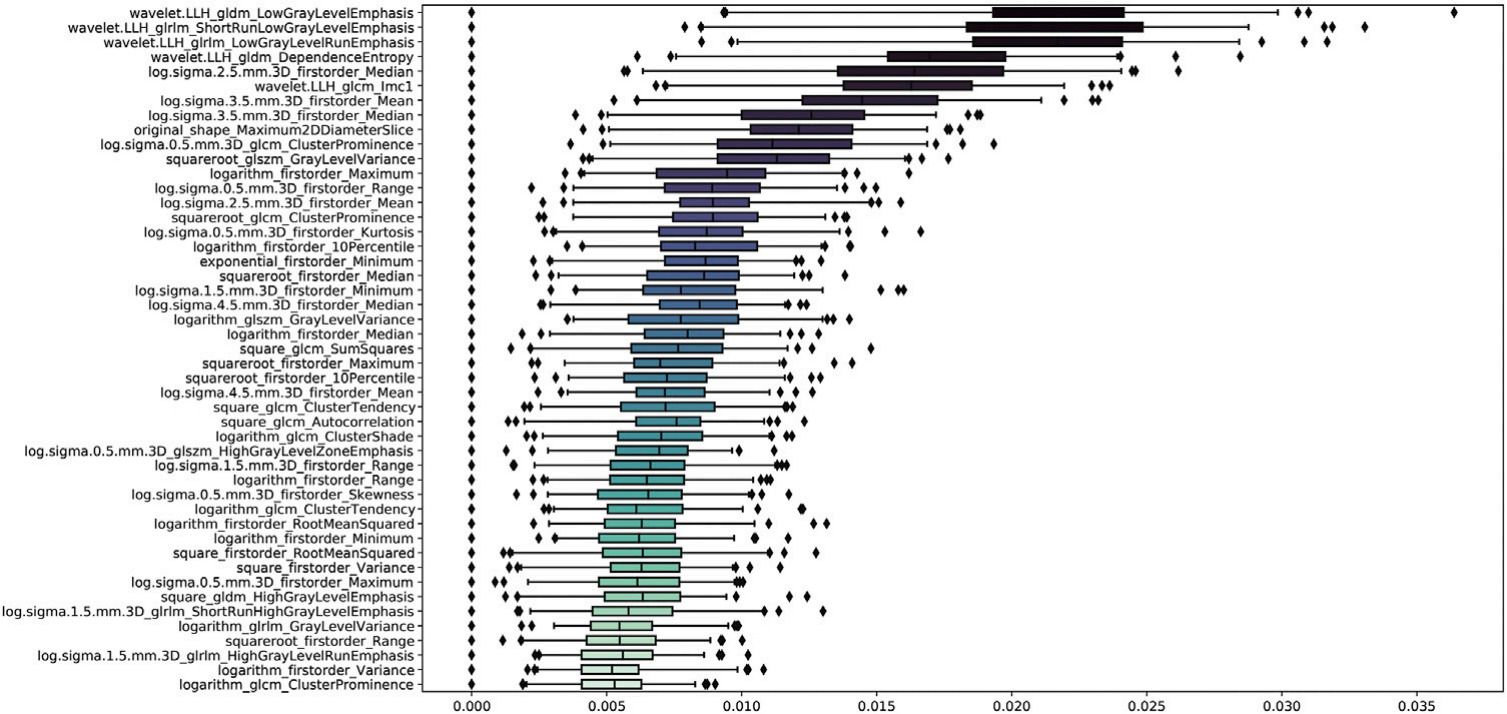
Importance ranking of 47 relevant radiomic features identified by Boruta algorithm in the training group. GLDM, gray level dependence matrix; GLRLM, gray level run length matrix; GLCM, gray level co-occurrence matrix; GLSZM, gray level size zone matrix.

**FIGURE 4:**
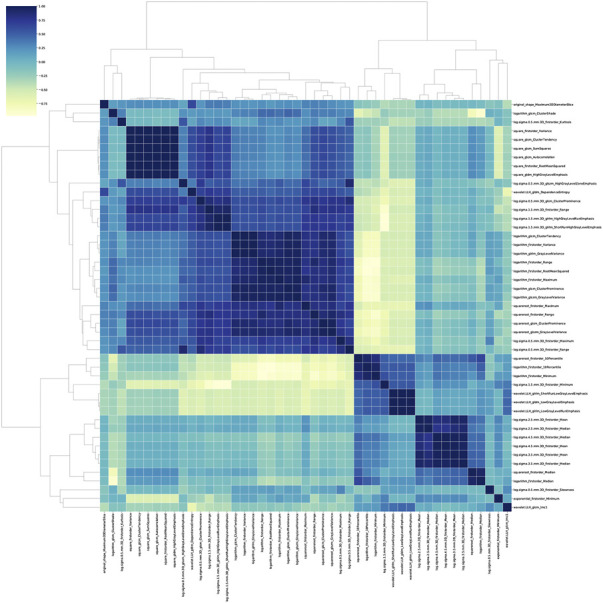
Correlation heatmap and dendrogram derived from hierarchical clustering in the training group. Boruta algorithm identified 8 different clusters derived from 47 important features. The darker color indicates higher correlation coefficient. GLDM, gray level dependence matrix; GLCM, gray level co-occurrence matrix; GLSZM, gray level size zone matrix; GLRLM, gray level run length matrix.

**FIGURE 5:**
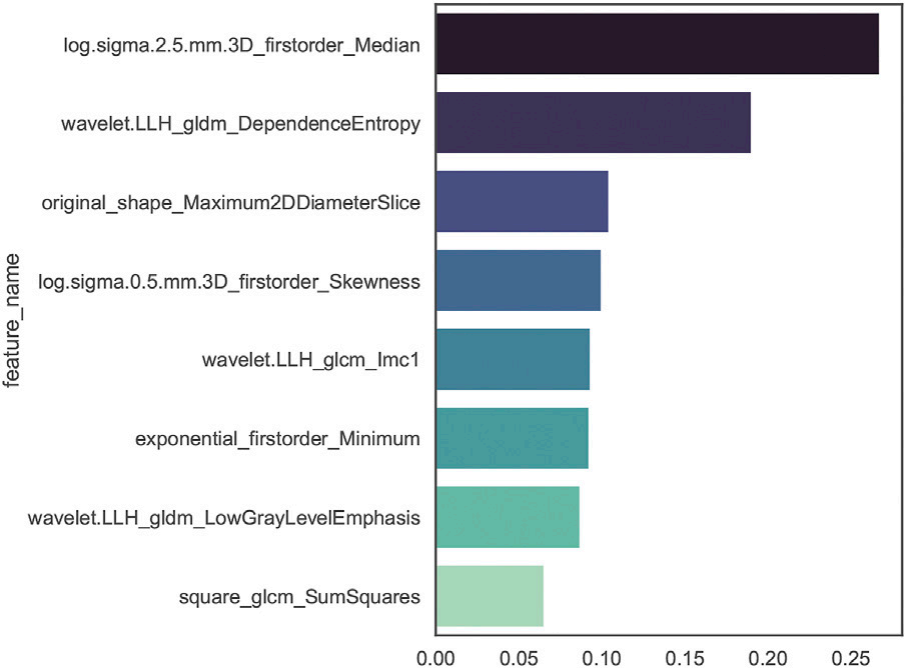
Importance ranking of the top-8 contributive features established by random forest algorithm of discriminating functional ischemia in the training group. The darker color indicates higher correlation. GLDM, gray level dependence matrix; GLCM, gray level co-occurrence matrix.

The stability analysis revealed 429 radiomic features with an intraclass correlation coefficient >0.8 out of 1691 features extracted from the training group. Boruta algorithm identified 47 candidate features which were highly associated with functional ischemia (Figure 3 and Supplementary Table S3). Hierarchical clustering demonstrated 8 distinct clusters of highly correlated radiomic features to functional ischemia (Figure 4). According to the feature importance, the most contributive feature from each of the 8 clusters was selected to establish the Rad-signature (Figure 5). The eight most contributive features included 4 texture features, 3 gray-level features, and 1 geometric feature. The correlation diagram and paired plots show minimal association among these top-8 contributive features (Supplementary Figure S2). The swarm plots show the distribution of radiomic features in the ischemia (CT-FFR ≤ 0.8) and nonischemic (CT-FFR > 0.8) groups (Supplementary Figure S3). The optimal cut-off value of Rad-signature to define functional ischemia was 0.202 according to the Youden index in the training group.

### Identifying functional ischemia of rad-signature, fat attenuation index, and anatomical CT

The performance of Rad-signature, FAI, and anatomical CT in identifying functional ischemia was evaluated at the vessel level. In the training group, the AUC of Rad-signature was 0.83 [95% confidence interval (CI): 0.78–0.87], significantly higher than 0.55 (0.49–0.62) of FAI (DeLong’s *p* < 0.001), but not higher than 0.79 (0.74–0.83) of anatomical CT (*p* = 0.175). The AUC of the combined model of Rad-signature and anatomical CT increased to 0.86 (0.82–0.90), higher than 0.83 (0.78–0.87) of only Rad-signature (*p* = 0.054). After adding Rad-signature to the traditional anatomical CT model, the net reclassification index was 0.25 (0.13–0.38, *p* < 0.001), which indicated the incremental value of Rad-signature in discriminating functional ischemia.

In the validation group, the AUC of Rad-signature was 0.82 (0.74–0.89), significantly higher than 0.55 (0.46–0.64) of FAI (*p* < 0.001), but not higher than 0.78 (0.71–0.85) of anatomical CT (*p* = 0.443). The AUC of the combined Rad-signature and anatomical CT model was 0.85 (0.79–0.91), significantly higher than that of FAI (*p* < 0.001) and anatomical CT (*p* = 0.012).

**FIGURE 6:**
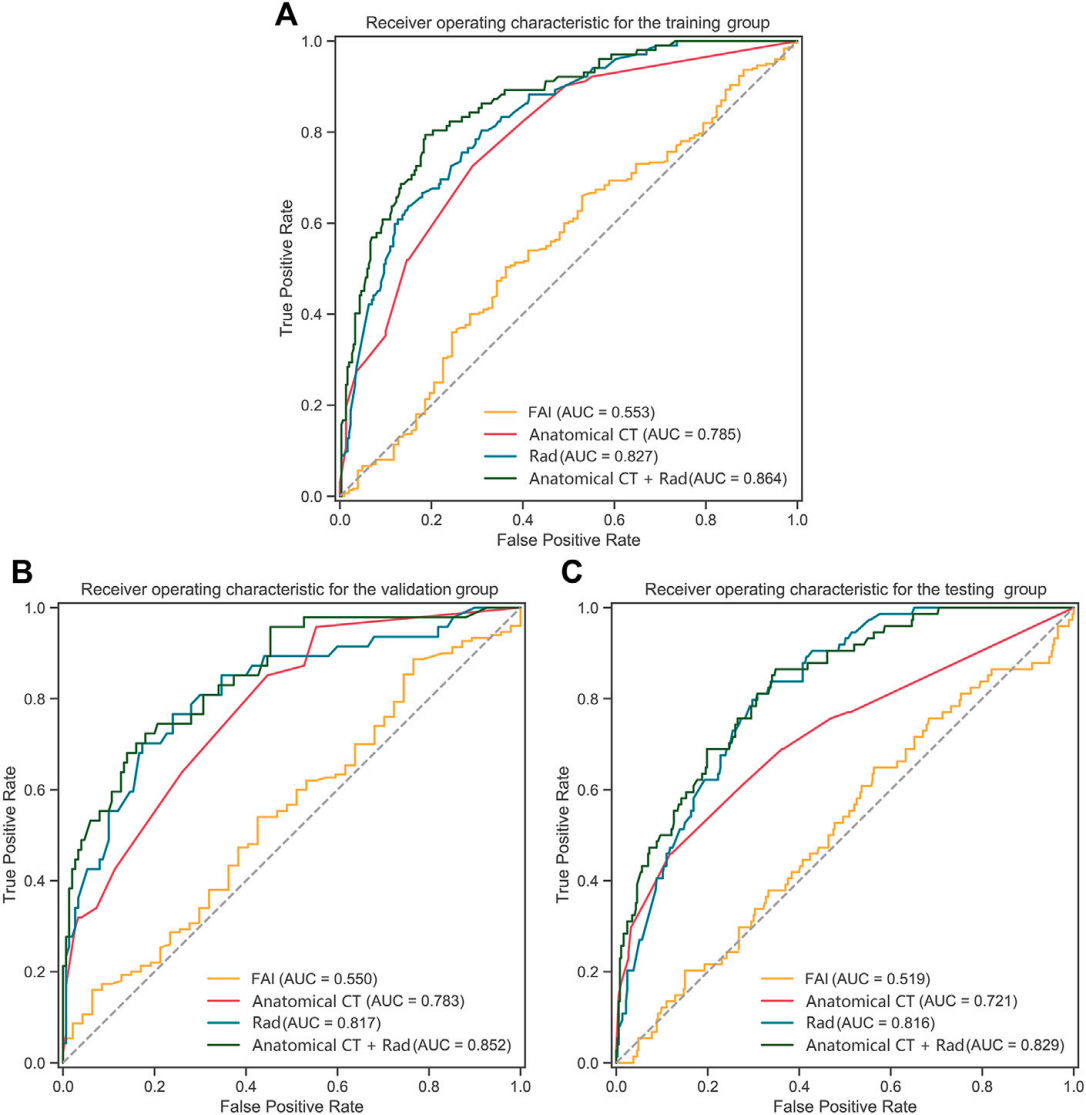
ROC curves of Rad-signature, FAI, anatomical CT and combined model (Rad-signature and anatomical CT) in **(A)** the training, **(B)** validation, and **(C)** testing groups. ROC, receiver operating characteristic; AUC, area under the ROC curve; Rad, Rad-signature; FAI, fat attenuation index.

In the testing group, the AUC of Rad-signature was 0.82 (0.77–0.86), significantly higher than 0.52 (0.45–0.59) of FAI (*p* < 0.001) and 0.72 (0.65–0.79) of anatomical CT (*p* = 0.017). The AUC of the combined Rad-signature and anatomical CT model was 0.83 (0.77–0.91), significantly higher than that of FAI or anatomical CT (*p* < 0.001). Table 3 and Figure 6 show the comparison of AUCs among the three groups**.** In addition, in the subgroup of obstructive CAD (stenosis ≥ 50%) identified by ICA (284 vessels in 95 patients), the AUC of Rad-signature was 0.76 (0.68–0.84), significantly higher than 0.53 (0.42–0.63) of FAI (*p* < 0.001) (Supplementary Table S4). The AUC of the combined Rad-signature and anatomical CT model was 0.80 (0.73–0.88), significantly higher than that of other models (*p* < 0.05). In the analysis of subgroups divided by vessels, the RCA, LAD, and LCx groups, the AUC of the combined model was significantly higher than that of FAI and anatomical CT in the three subgroups (all *p* < 0.05) and showed an incremental value on the basis of Rad-signature (Supplementary Table S5).

**TABLE 3:** Performance metrics of all models at the vessel level.

Model	AUC	95% CI	Accuracy	95%CI	Sensitivity	Specificity	*p*-Value
Training group							
Rad-signature	0.83	0.78–0.87	0.72	0.67–0.76	0.80	0.69	0.020
Anatomical CT	0.79	0.74–0.83	0.71	0.67–0.76	0.73	0.71	**<0.001**
FAI	0.55	0.49–0.62	0.46	0.41–0.51	0.36	0.50	**<0.001**
Combined model (Rad-signature and anatomical CT)	0.86	0.82–0.90	0.81	0.77–0.85	0.79	0.81	N/A
Validation group							
Rad-signature	0.82	0.74–0.89	0.71	0.64–0.77	0.81	0.67	0.098
Anatomical CT	0.78	0.71–0.85	0.72	0.65–0.78	0.64	0.74	**0.012**
FAI	0.55	0.46–0.64	0.45	0.38–0.52	0.51	0.43	**<0.001**
Combined model (Rad-signature and anatomical CT)	0.85	0.79–0.91	0.82	0.76–0.87	0.68	0.86	N/A
Testing group							
Rad-signature	0.82	0.77–0.86	0.69	0.64–0.73	0.82	0.66	0.531
Anatomical CT	0.72	0.65–0.79	0.71	0.66–0.75	0.61	0.73	**<0.001**
FAI	0.52	0.45–0.59	0.51	0.46–0.56	0.54	0.50	**<0.001**
Combined model (Rad-signature and anatomical CT)	0.83	0.77–0.91	0.78	0.74–0.82	0.66	0.80	N/A

*p*-Values represents the difference between the AUC of the model and combined model (Rad-signature and anatomic CT).

Bold values signify statistical significance.

AUC, area under the ROC curve; CI, confidence interval; Anatomical CT, coronary stenosis grade on CCTA; FAI, fat attenuation index.

In the CCTA-invasive FFR group, at the vessel level, using invasive FFR as the gold standard, the accuracy of CT-FFR was 85.4%, and the AUC of CT-FFR was 0.928 (0.85–1.00). The AUC of Rad-signature in the 4-fold cross-validation was 0.81, 0.86, 0.84, and 0.83, and the mean AUC was 0.83 ± 0.02. Supplementary Figure S4 shows the AUCs of Rad-signature in the cross-validation.

### Influential factors of functional ischemia

For all 350 patients in this study, at the patient level, univariate logistic regression showed that sex, left ventricular mass derived from CCTA images (LVM-CT), Rad-signature of LAD (Rad-LAD), Rad-signature of LCx (Rad-LCx), the mean Rad-signature of RCA, LAD, and LCx (Rad-mean), and anatomical CT were significantly associated with functional ischemia (*p* < 0.05). Multivariate logistic regression showed that Rad-LAD [OR = 1.7 (95% CI: 1.1–2.6), *p* = 0.018] and anatomical CT [OR = 3.5 (2.1–6.0), *p* < 0.01] were independent influential factors of functional ischemia.

The patients in the testing group (*n* = 150) were divided into two subgroups according to the ICA results, i.e., 95 (63.3%) patients with obstructive CAD (stenosis ≥ 50%) and 55 (36.7%) non-obstructive (<50%). In the obstructive subgroup, univariate logistic regression showed that age, LVM-CT, Rad-LAD, Rad-LCx, and Rad-mean were significantly associated with functional ischemia (all *p* < 0.05). Multivariate logistic regression showed that Rad-LAD [OR = 2.45 (95% CI: 1.08–6.27), *p* = 0.042] independently associated with functional ischemia. In the nonobstructive subgroup, univariate logistic regression showed that FAI-LAD (*p* = 0.026) and Rad-LCx (*p* = 0.067) were strongly associated with functional ischemia. Multivariate logistic regression showed that FAI-LAD [OR = 1.1 (1.0–1.2), *p* = 0.041] independently associated with functional ischemia. Tables 4, 5 show the logistic regression results**.**

**TABLE 4:** Logistic regression analysis of discriminating coronary functional ischemia in the whole study population at the patient level.

Variables	Univariate analysis		Multivariate analysis	
	OR (95% CI)	*p*-Value	OR (95% CI)	*p*-Value
Sex	0.49 (0.32–0.76)	**0.001**	0.78 (0.38–1.62)	0.506
Age	0.98 (0.96–1.01)	0.181		
Hypertension	0.96 (0.63–1.47)	0.861		
Diabetes	1.25 (0.8–1.94)	0.326		
Hyperlipemia	1.42 (0.92–2.22)	0.115		
Smoking	1.25 (0.78–2.01)	0.359		
Family history of CAD	0.83 (0.38–1.77)	0.628		
BMI	0.97 (0.91–1.03)	0.282		
LVM-CT	1.01 (1.00–1.01)	**0.023**	1.01 (1.00–1.01)	0.106
Rad-LAD	1.99 (1.57–2.56)	**<0.001**	1.72 (1.13–2.64)	**0.018**
Rad-RCA	3.17 (0.28–37.65)	0.352		
Rad-LCx	1.49 (1.19–1.88)	**<0.001**	1.22 (0.85–1.74)	0.274
Rad-mean	2.02 (1.59–2.61)	**<0.001**	1.25 (0.76–2.09)	
FAI-LAD	1.01 (0.98–1.03)	0.652		
FAI-RCA	1.02 (1.00–1.04)	0.097	1.00 (0.94–1.03)	0.980
FAI-LCx	1.00 (0.98–1.02)	0.878		
Anatomic CT	3.74 (2.32–6.13)	**<0.001**	3.52 (2.10–6.04)	**<0.001**

OR, odds ratio; CI, confidence interval; CAD, coronary artery disease; BMI, body mass index; LVM-CT, left ventricular mass on CT; Rad-LAD, rad-score of left anterior descending; Rad-RCA, rad-score of right coronary artery; Rad-LCx, rad-score of left circumflex; Rad-mean, the mean rad-score of LAD, RCA and LCx; FAI-LAD, fat attenuation index of LAD; FAI-RCA, FAI of RCA; FAI-LCx, FAI of LCx; Anatomic CT, coronary stenosis grade on CCTA.

Bold values signify statistical significance.

**TABLE 5:** Logistic regression analysis of discriminating coronary functional ischemia in subgroups divided by invasive coronary angiography in the testing group at the patient level.

Variables	Non-obstructive (stenosis < 50%) (*n* = 55)				Obstructive (stenosis ≥ 50%) (*n* = 95)			
	Univariate analysis		Multivariate analysis		Univariate analysis		Multivariate analysis	
	OR (95% CI)	*p*-Value	OR (95% CI)	*p*-Value	OR (95% CI)	*p*-Value	OR (95% CI)	*p*-Value
Sex	0.37 (0.10–1.24)	0.117			0.31 (0.12–0.74)	**0.01**	1.35 (0.27–7.26)	0.717
Age	0.98 (0.91–1.06)	0.652			0.95 (0.9–0.99)	**0.021**	0.94 (0.88–1.00)	0.054
Hypertension	0.84 (0.25–2.84)	0.782			0.83 (0.37–1.86)	0.648		
Diabetes	0.66 (0.13–2.58)	0.571			0.81 (0.31–2.09)	0.668		
Hyperlipemia	1.31 (0.39–4.38)	0.656			2.37 (0.9–6.6)	0.086	2.09 (0.64–7.22)	0.227
Smoking	1.5 (0.39–5.38)	0.538			1.88 (0.8–4.48)	0.148		
Family history of CAD	1.36 (0.06–15.27)	0.809			0.26 (0.01–1.85)	0.238		
BMI	0.92 (0.75–1.1)	0.37			1.03 (0.91–1.16)	0.671		
LVMCT.	1.01 (0.99–1.03)	0.241			1.01 (1–1.03)	**0.031**	1.01 (0.99–1.03)	0.267
Rad_LAD	1.60 (0.86–3.17)	0.15			2.07 (1.32–3.46)	**0.003**	2.45 (1.08–6.27)	**0.042**
Rad_RCA	0.78 (0.39–1.43)	0.447			0.88 (0.56–1.33)	0.566		
Rad_LCx	1.74 (0.97–3.26)	0.067	1.65 (0.89–3.21)	0.117	1.72 (1.10–2.87)	**0.025**	1.71 (0.80–4.30)	0.2
Rad_mean	1.54 (0.85–2.94)	0.166			1.92 (1.23–3.17)	**0.006**	0.84 (0.25–2.24)	0.739
FAI_LAD	1.11 (1.02–1.22)	**0.026**	1.10 (1.01–1.22)	**0.041**	1 (0.96–1.04)	0.894		
FAI_RCA	1.04 (0.98–1.12)	0.236			1.02 (0.98–1.07)	0.359		
FAI_LCx	1.02 (0.93–1.11)	0.711			1.01 (0.97–1.04)	0.745		

OR, odds ratio; CI, confidence interval; CAD, coronary artery disease; BMI, body mass index; LVM-CT, left ventricular mass on CT; Rad-LAD, rad-score of left anterior descending; Rad-RCA, rad-score of right coronary artery; Rad-LCx, rad-score of left circumflex; Rad-mean, the mean rad-score of LAD, RCA, and LCx; FAI, fat attenuation index.

Bold values signify statistical significance.

Additionally, in the subgroup of patients with single-vessel disease, Rad-LAD, Rad-mean, and FAI-RCA were independently associated with functional ischemia. In the subgroup of patients with multiple vessel disease, Rad-LAD, Rad-LCx, and Rad-mean were independently associated with functional ischemia (Supplementary Table S6).

## Discussion

Based on the comprehensive imaging features of PCT, we successfully established a radiomics signature (Rad-signature) to discriminate coronary functional ischemia. The AUC of the Rad-signature reached 0.82 in the validation and testing groups, which was significantly higher than that of FAI and numerically higher than anatomical CT but not statistically significant. The combined model of Rad-signature and anatomical CT increased the AUC compared with only anatomical CT, so adding Rad-signature has incremental value in discriminating functional ischemia. We also ensured that Rad-signature provides a good discrimination ability of standard functional ischemia (invasive FFR ≤ 0.8).

Coronary stenosis degree on CCTA is a widely-used indicator in diagnosing CAD with high accuracy and negative predictive value (Meijboom et al., 2008; Garg et al., 2016). However, conventional CCTA only provides morphological information but does not provides hemodynamic significance and lesion-specific ischemia, the functional ischemia (Park et al., 2012). In recent years, researchers have realized the inconsistency between morphologic stenosis and functional ischemia. Tonino et al. reported that 65% of the patients with moderate coronary stenosis and 20% with severe stenosis were not functionally significant (Tonino et al., 2010). Park et al. conducted a prospective study and found that the frequency of visual-functional mismatch was 40% between coronary angiography and FFR (Park et al., 2012). Furthermore, Pijls et al. found that the patients with CAD benefited more from FFR-guided revascularization strategies than morphological assessment by standard angiography, and 2-years mortality and incidence of myocardial infarction were significantly reduced (Pijls et al., 2010). CT-FFR noninvasively measures lumen blood flow, but it often depends on dedicated software, and sometimes the remote processing results are not timely, which limits the wide application of CT-FFR. The establishment of Rad-signature can be performed with an on-site workstation or personal computer, thus Rad-signature may be a practical and economical indicator. Since clinical decision-making should depend on the coronary functional significance, our study provides new evidence for the diagnosis and treatment of CAD by mining the functional correlation from anatomical CT.

Radiomics can extract a large number of imaging features from CCTA from a computational point of view. Meanwhile, machine learning can effectively select valuable information from numerous features and establish predictive models (Zhang et al., 2021). In our study, the Rad-signature was a powerful predictor and independent influential factor of functional ischemia. It derived from the eight most contributive radiomic features extracted from PCT on CCTA images, including 4 texture, 3 gray-level, and 1 geometric feature. Half of these contributive features were texture features, which were wavelet and log transformation based on the Gray-Level Co-occurrence Matrix which describes the spatial relationships of pixel pairs or voxel pairs with predefined gray intensity, and Gray Level Dependence Matrix which describes the grayscale relationship between the central pixel or the voxel and its neighborhood. The texture features may reflect the heterogeneity of PCT. The gray level features may reflect the intensity of PCT. Therefore, the main components of Rad-signature are not only the intensity information similar to FAI, but also the image heterogeneity information beyond the traditional image analysis standards. Similarly, Oikonomou et al. discussed that the radiomic features of PCAT derived from CCTA were highly associated with pathologically confirmed fibrosis and microvascular remodeling, and can differentiate patients with acute myocardial infarction and stable CAD, because they capture the spatial shifts in composition and lipid content of PCAT (Oikonomou et al., 2019). Vascular inflammation leads not only to plaque formation and lumen stenosis, but also to endothelial dysfunction and impaired vasodilation (Margaritis et al., 2013; Antonopoulos et al., 2017), which may decrease distal flow reserve and cause functional ischemia.

We also conducted a subgroup analysis and evaluated the influential factors of functional ischemia. In the subgroup of obstructive CAD, Rad-LAD was an independent influential factor of functional ischemia. It suggests that the radiomic phenotype may be associated with pericoronary inflammation and easier to be captured in patients with obstructive CAD. In the nonobstructive subgroup, FAI-LAD rather than Rad-signature was an independent factor of functional ischemia. With the development of coronary atherosclerosis, the histological structure of PCT changes accordingly. Therefore, Rad-signature may be suitable for patients with moderate and severe coronary stenosis and FAI may be applicable for those with minimal and mild stenosis. Similarly, Antonopoulos et al. (2017) reported that FAI can be used to detect vascular inflammation at an early stage and change dynamically with the status of inflammation.

here are limitations. First, our study used a time-independent testing group instead of an external testing group. Multiple CT equipment enhanced the robustness of the model, but if external testing is adopted, the difference in imaging features caused by different CT devices between the two centers may interfere with the exploration of the relationship between PCT and functional ischemia. Second, we evaluated the ability of Rad-signature in discriminating functional ischemia defined as CT-FFR ≤ 0.8. Although CT-FFR ≤ 0.8 is widely considered coronary functional significance, noninvasive CT-FFR does not directly measure blood flow. Different reference standards may lead to a different selection of radiomic features, which means that further study is necessary to refine and calibrate the Rad-signature model. Furthermore, preliminary exploration in the CCTA-invasive FFR group suggested that Rad-signature may have a good ability in discriminating standard functional ischemia. However, more cases were needed to further validate the results.

## Conclusion

The machine-learning-derived radiomics model of Rad-signature of PCT showed a good ability in discriminating coronary functional ischemia. It may potentially become a noninvasive, fast, and economical indicator to screen functional ischemia before expensive invasive examinations. The combined model demonstrated the incremental value of Rad-signature to anatomical CT, rather than the superiority of Rad-signature alone in discriminating functional ischemia, which may help identify high-risk patients.

## Data Availability

The raw data supporting the conclusions of this article will be made available by the authors, without undue reservation.
