# Supplementary material for: Machine-learning-derived radiomics signature of pericoronary tissue in coronary CT angiography associates with functional ischemia
Source: Front Physiol. 2022 Sep 26;13:980996. doi: 10.3389/fphys.2022.980996 (PMC9550214; doi:10.3389/fphys.2022.980996)
Supplement: Supplementary file 1 [file Table1.DOCX]

| **CT equipment** | **Hospital-1** | | | |  | **Hospital-2** | |
| --- | --- | --- | --- | --- | --- | --- | --- |
|  | **Siemens Somatom Force** | **GE Revolution CT** | **GE Discovery750 HD** | **TOSHIBA Aquilion ONE** |  | **Siemens Somatom Definition Flash** | **Siemens Somatom Force** |
| Detector collimation, mm | 192×0.6 | 256×0.625 | 64×0.625 | 320×0.5 |  | 128×0.6 | 192×0.6 |
| Gantry rotation time, ms | 280 | 280 | 350 | 275 |  | 280 | 280 |
| Field of view, mm | 500 | 320 | 320 | 320 |  | 500 | 500 |
| Reconstruction thickness, mm | 0.75 | 0.63 | 0.63 | 0.5 |  | 0.6 | 0.75 |
| Slice increment, mm | 0.75 | 0.63 | 0.63 | 0.5 |  | 0.6 | 0.75 |
| Reconstruction kernel | bv40d/3 | standard | standard | FC43 |  | I26f/2 | bv40d/3 |

**Supplementary Table 1. CCTA acquisition and reconstruction parameters**

**Supplementary Table 2. Statistic packages list for this study.**

| **Statistical analysis** | **Packages** | **Version** |
| --- | --- | --- |
| Random Forest | ‘scikit-learn’ in Python | 0.13.2 |
| Grid Search with Cross validation | ‘scikit-learn’ in Python | 0.13.2 |
| Feature selection: Boruta | ‘Boruta’ in Python | 0.3 |
| Feature selection: Correlation | ‘sicpy’ in Python | 1.6.0 |
| Feature selection: Stability test: intraclass correlation coefficient | ‘irr’ in R | 0.84.1 |
| multivariate logistic regression | ‘rms’ in R | 6.0-1 |
| Delong Test and 95%CI for ROC curve | ‘pROC’ in R | 1.16.2 |
| Net reclassification index | 'PredictABEL' in R | 1.2-4 |

**Supplementary Table 3. 47 radiomic features highly associated with functional ischemia identified by Boruta algorithm from 429 stable features.**

| **Radiomic feature name** | **Mean importance** |
| --- | --- |
| wavelet.LLH_gldm_LowGrayLevelEmphasis | 0.02133263 |
| wavelet.LLH_glrlm_ShortRunLowGrayLevelEmphasis | 0.020953771 |
| wavelet.LLH_glrlm_LowGrayLevelRunEmphasis | 0.020666204 |
| wavelet.LLH_gldm_DependenceEntropy | 0.016807744 |
| log.sigma.2.5.mm.3D_firstorder_Median | 0.01629396 |
| wavelet.LLH_glcm_Imc1 | 0.015676491 |
| log.sigma.3.5.mm.3D_firstorder_Mean | 0.014408687 |
| log.sigma.3.5.mm.3D_firstorder_Median | 0.012128655 |
| original_shape_Maximum2DDiameterSlice | 0.011881218 |
| log.sigma.0.5.mm.3D_glcm_ClusterProminence | 0.011278171 |
| squareroot_glszm_GrayLevelVariance | 0.010968708 |
| logarithm_firstorder_Maximum | 0.009145968 |
| log.sigma.0.5.mm.3D_firstorder_Range | 0.008909514 |
| log.sigma.2.5.mm.3D_firstorder_Mean | 0.008843523 |
| squareroot_glcm_ClusterProminence | 0.008764988 |
| log.sigma.0.5.mm.3D_firstorder_Kurtosis | 0.00862474 |
| logarithm_firstorder_10Percentile | 0.008609881 |
| exponential_firstorder_Minimum | 0.008222986 |
| squareroot_firstorder_Median | 0.008199854 |
| log.sigma.1.5.mm.3D_firstorder_Minimum | 0.008160164 |
| log.sigma.4.5.mm.3D_firstorder_Median | 0.00813073 |
| logarithm_glszm_GrayLevelVariance | 0.007944844 |
| logarithm_firstorder_Median | 0.007703782 |
| square_glcm_SumSquares | 0.007467398 |
| squareroot_firstorder_Maximum | 0.007350381 |
| squareroot_firstorder_10Percentile | 0.007233587 |
| log.sigma.4.5.mm.3D_firstorder_Mean | 0.007183757 |
| square_glcm_ClusterTendency | 0.007181037 |
| square_glcm_Autocorrelation | 0.007073379 |
| logarithm_glcm_ClusterShade | 0.006886918 |
| log.sigma.0.5.mm.3D_glszm_HighGrayLevelZoneEmphasis | 0.006677157 |
| log.sigma.1.5.mm.3D_firstorder_Range | 0.006556553 |
| logarithm_firstorder_Range | 0.006488961 |
| log.sigma.0.5.mm.3D_firstorder_Skewness | 0.006468101 |
| logarithm_glcm_ClusterTendency | 0.006339896 |
| logarithm_firstorder_RootMeanSquared | 0.006310308 |
| logarithm_firstorder_Minimum | 0.006200785 |
| square_firstorder_RootMeanSquared | 0.006192051 |
| square_firstorder_Variance | 0.006180626 |
| log.sigma.0.5.mm.3D_firstorder_Maximum | 0.006153667 |
| square_gldm_HighGrayLevelEmphasis | 0.006144105 |
| log.sigma.1.5.mm.3D_glrlm_ShortRunHighGrayLevelEmphasis | 0.006003816 |
| logarithm_glrlm_GrayLevelVariance | 0.005662036 |
| squareroot_firstorder_Range | 0.0055378 |
| log.sigma.1.5.mm.3D_glrlm_HighGrayLevelRunEmphasis | 0.005518761 |
| logarithm_firstorder_Variance | 0.005280077 |
| logarithm_glcm_ClusterProminence | 0.005153619 |

**Supplementary Table 4. Performance metrics of models in subgroup of obstructive CAD.** P-values represents the difference between the AUC of the model and combined model (Rad-signature and anatomic CT). Bold values signify statistical significance. AUC, area under the ROC curve; CI, confidence interval; Anatomic CT, coronary stenosis grade on CCTA; FAI, fat attenuation index.

| **Model** | **AUC** | **95%CI** | **p-value** |
| --- | --- | --- | --- |
| Rad-signature | 0.76 | 0.68-0.84 | **0.049** |
| Anatomical CT | 0.71 | 0.61-0.80 | **0.029** |
| FAI | 0.53 | 0.42-0.63 | **<0.001** |
| Combined model  (Rad-signature and anatomical CT) | 0.80 | 0.73-0.88 | N/A |

**Supplementary Table 5. Performance metrics of models in subgroups divided by vessels (RCA, LAD and LCx).** P-values represents the difference between the AUC of the model and combined model (Rad-signature and anatomic CT). Bold values signify statistical significance. AUC, area under the ROC curve; CI, confidence interval; Anatomic CT, coronary stenosis grade on CCTA; FAI, fat attenuation index.

| **Model** | **RCA (n=150)** | | |  | **LAD (n=150)** | | |  | **LCx (n=147)** | | |
| --- | --- | --- | --- | --- | --- | --- | --- | --- | --- | --- | --- |
|  | **AUC** | **p-value** | |  | **AUC** | **p-value** | |  | **AUC** | **p-value** | |
| Rad-signature | 0.77(0.70-0.85) | | 0.44 |  | 0.71(0.65-0.76) | | **<0.001** |  | 0.82(0.76-0.88) | | 0.362 |
| Anatomical CT | 0.70(0.62-0.79) | | **<0.001** |  | 0.73(0.67-0.78) | | **0.006** |  | 0.73(0.65-0.82) | | **<0.001** |
| FAI | 0.57(0.48-0.65) | | **<0.001** |  | 0.52(0.46-0.58) | | **<0.001** |  | 0.53(0.43-0.63) | | **<0.001** |
| Combined model (Rad-signature and anatomical CT) | 0.80(0.73-0.87) | | N/A |  | 0.78(0.73-0.83) | | N/A |  | 0.85(0.79-0.91) | | N/A |

**Supplementary Table 6. Logistic regression analysis of discriminating coronary functional ischemia in subgroups divided by single versus multiple vessel CAD in testing group at patient level.** OR, odds ratio; CI, confidence interval; CAD, coronary artery disease; BMI, body mass index; LVM-CT, left ventricular mass on CT; Rad-LAD, rad-score of left anterior descending; Rad-RCA, rad-score of right coronary artery; Rad-LCx, rad-score of left circumflex; Rad-mean, the mean rad-score of LAD, RCA, and LCx; FAI, fat attenuation index. Bold values signify statistical significance.

| **Variables** | **Single-vessel disease (n=53)** | | | |  | **Multi-vessel disease (n=82)** | | | |
| --- | --- | --- | --- | --- | --- | --- | --- | --- | --- |
|  | **Univariate analysis** | | **Multivariate analysis** | |  | **Univariate analysis** | | **Multivariate analysis** | |
|  | **OR (95% CI)** | **p-value** | **OR (95% CI)** | **p-value** |  | **OR (95% CI)** | **p-value** | **OR (95% CI)** | **p-value** |
| Sex | 0.37(0.17-0.77） | **0.009** | 0.92(0.24-3.66) | 0.901 |  | 0.64(0.36-1.12) | 0.117 | 0.96(0.92-0.99) | **0.016** |
| Age | 0.98（0.94-1.02） | 0.295 |  |  |  | 0.97(0.94-1.00) | 0.065 |  |  |
| Hypertension | 0.81（0.39-1.65） | 0.558 |  |  |  | 0.87(0.50-1.53) | 0.649 |  |  |
| Diabetes | 0.89（0.39-1.96） | 0.768 |  |  |  | 1.19(0.69-2.09) | 0.518 |  |  |
| Hyperlipemia | 1.1(0.52-2.29) | 0.797 |  |  |  | 1.79(1.00-3.23) | 0.051 | 1.43(0.76-2.71) | 0.264 |
| Smoking | 3.24(1.48-5.57) | **0.004** | 2.16(0.78-6.18) | 0.14 |  | 0.72(0.39-1.32) | 0.291 |  |  |
| Family history of CAD | 1.59(0.43-5.57) | 0.467 |  |  |  | 0.56(0.21-1.48) | 0.243 |  |  |
| BMI | 0.9(0.8-1.01) | 0.078 | 0.92(0.80-1.04) | 0.198 |  | 1.00(0.92-1.08) | 0.955 |  |  |
| LVM.CT. | 1.01(1-1.02) | 0.118 |  |  |  | 1.01(1.00-1.01) | 0.12 |  |  |
| Rad_LAD | 2.22(1.47-3.52) | **<0.001** | 2.02(1.11-3.82) | **0.025** |  | 1.81(1.35-2.47) | **<0.001** | 1.52(0.92-2.54) | 0.106 |
| Rad_RCA | 1.05(0.73-1.49) | 0.808 |  |  |  | 1.11(0.84-1.48) | 0.478 |  |  |
| Rad_LCx | 1.14(0.8-1.62) | 0.472 |  |  |  | 1.74(1.27-2.47) | **0.001** | 1.50(0.96-2.41) | 0.081 |
| FAI_LAD | 1.02(0.98-1.06) | 0.25 |  |  |  | 0.99(0.97-1.02) | 0.69 |  |  |
| FAI_RCA | 1.03(1-1.07) | 0.096 | 1.18(0.66-2.11) | 0.579 |  | 1.01(0.98-1.04) | 0.533 |  |  |
| FAI_LCx | 0.99(0.95-1.02) | 0.597 |  |  |  | 1.01(0.98-1.04) | 0.469 |  |  |
| Rad_mean | 1.84(1.26-2.77) | **0.002** | 1.18(0.66-2.11) | 0.579 |  | 2.01(1.48-2.80) | **<0.001** | 1.21(0.66-2.29) | 0.535 |

**Supplementary Figure 1.** A representative case of Rad-signature establishment. CCTA: coronary computed tomography angiography. CT-FFR: CT-derived fractional flow reserve. pLAD: proximal left anterior descending. FAI: fat attenuation index.PCT: pericoronary tissue.

**Supplementary Figure** **2.** Correlation diagram of the top-8 contributive features in training cohort. The darker indicted higher correlation. GLDM = gray level dependence matrix; GLCM = gray level co-occurrence matrix.

**Supplementary Figure 3.** Swam plots shows the distribution of the top-8 contributive features in the training cohort between the functional ischemia(blue) and non-ischemia(red) groups. GLDM, gray level dependence matrix; GLCM = gray level co-occurrence matrix.

**Supplementary Figure 4.** ROC curves of Rad-signature in the invasive FFR group by cross validation. ROC, receiver operating characteristic; AUC, area under the ROC curve.
