# Supplementary figures and images for: Machine-learning-derived radiomics signature of pericoronary tissue in coronary CT angiography associates with functional ischemia
Source: Front Physiol. 2022 Sep 26;13:980996. doi: 10.3389/fphys.2022.980996 (PMC9550214; doi:10.3389/fphys.2022.980996)

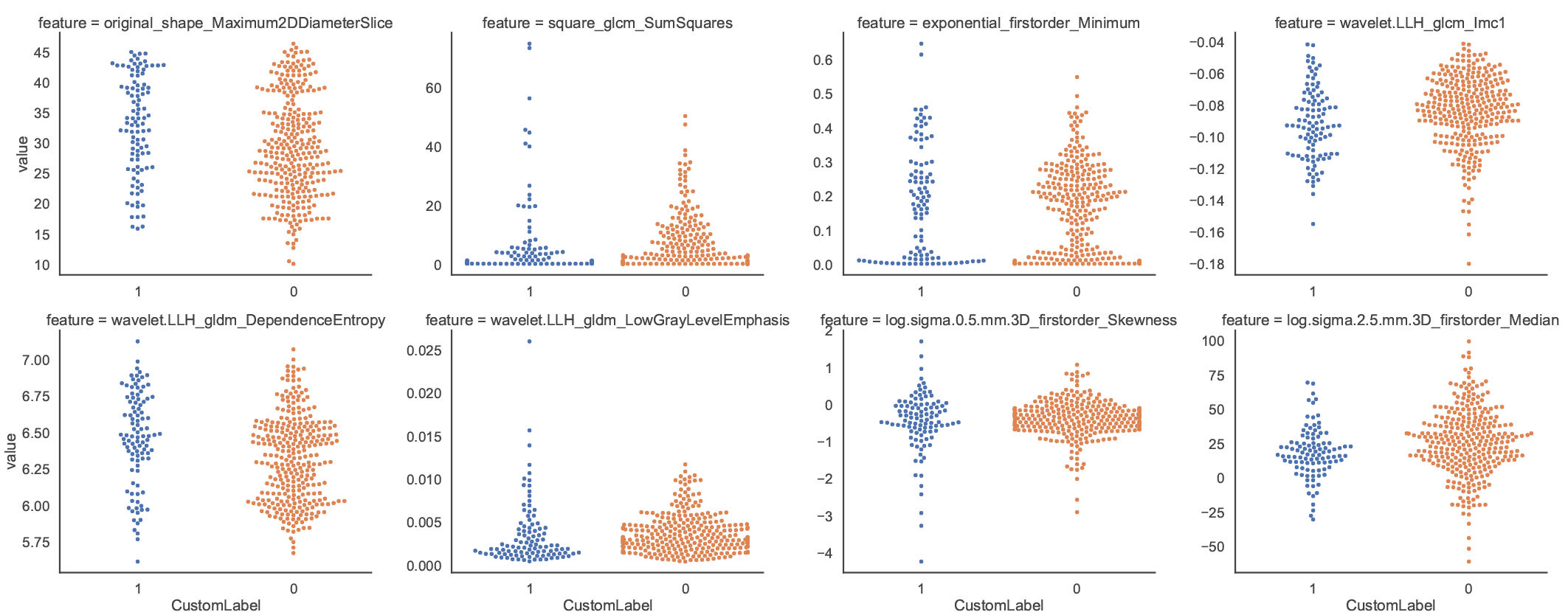

Supplement: Supplementary file 2 [file Image3.TIF]

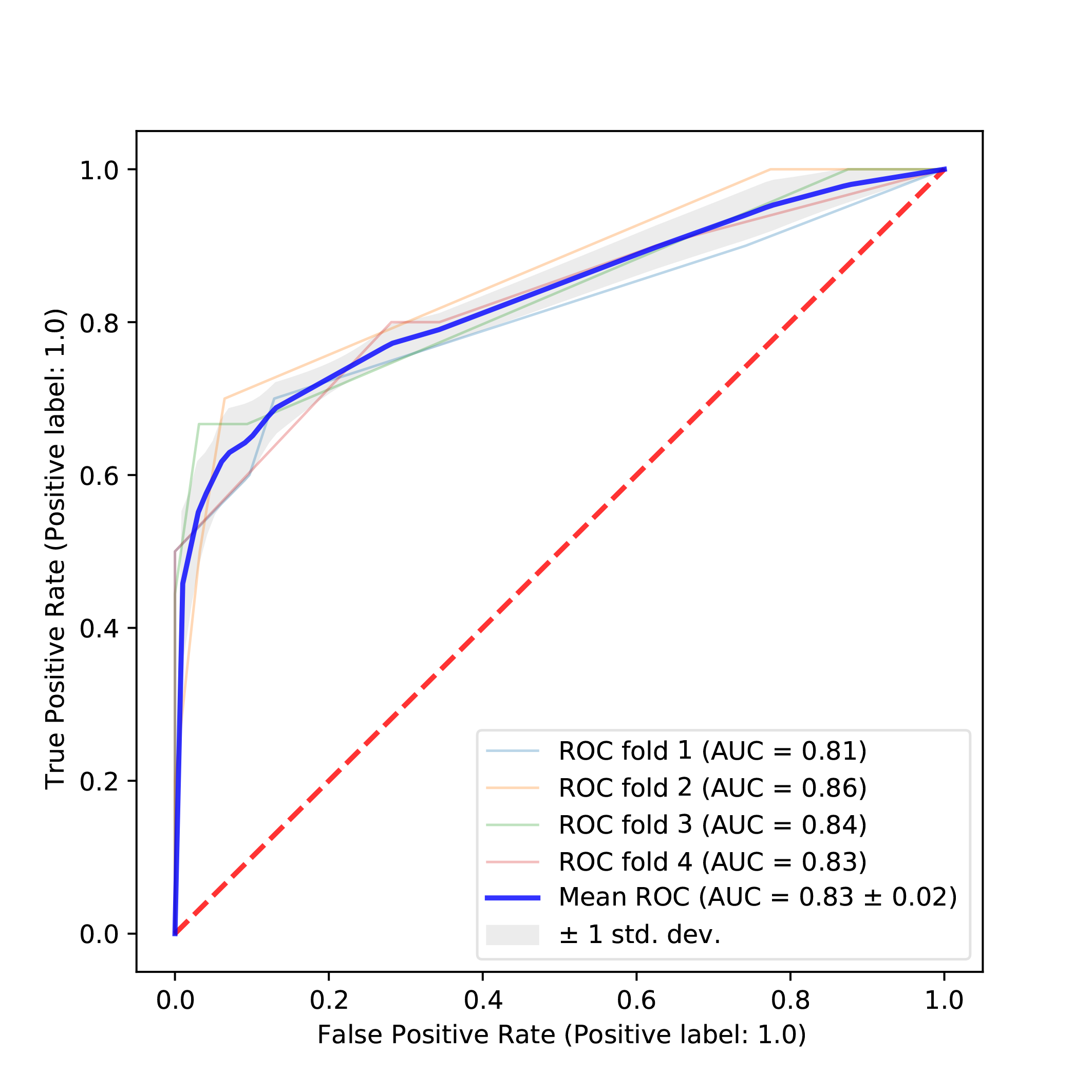

Supplement: Supplementary file 3 [file Image4.TIF]

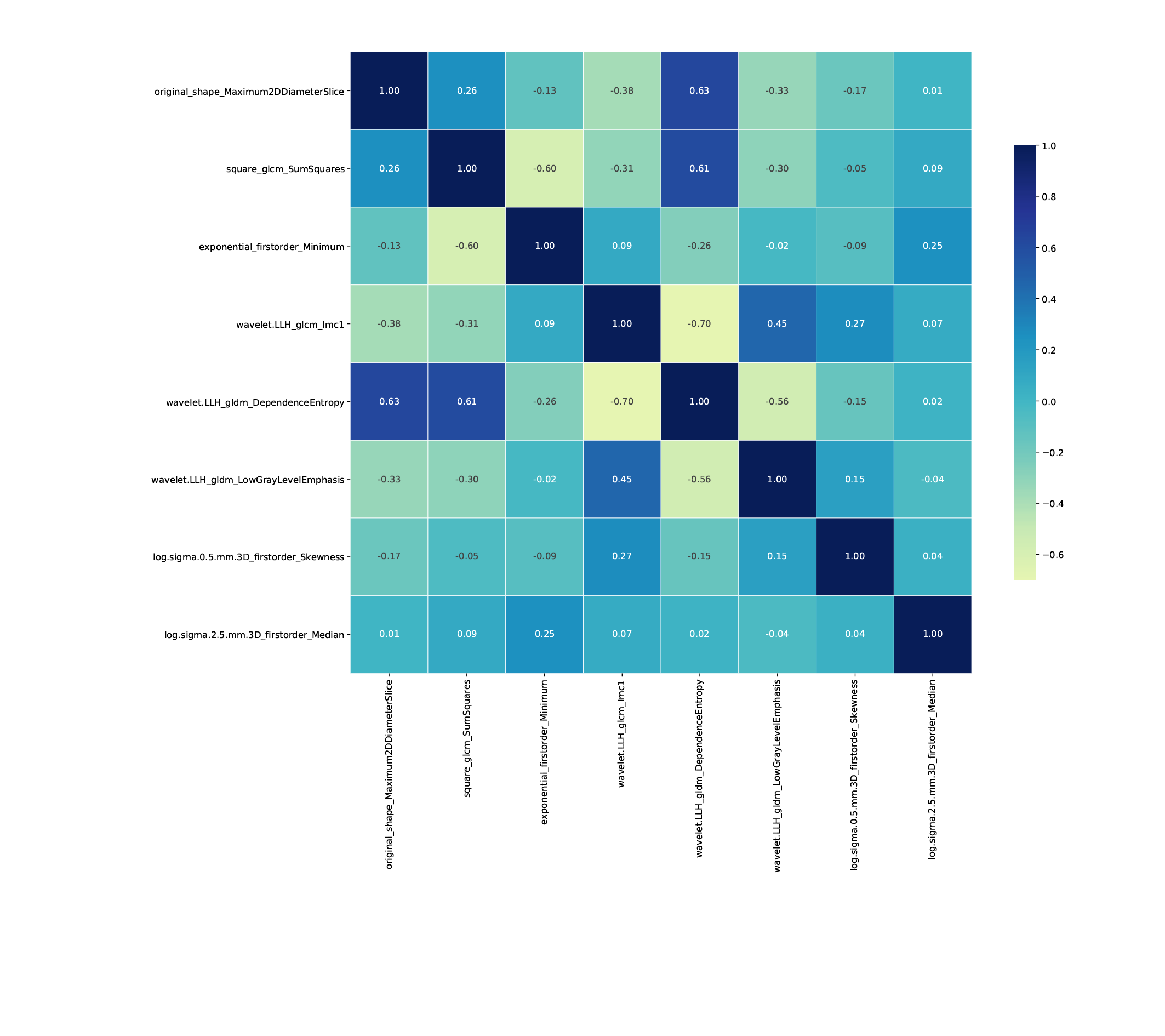

Supplement: Supplementary file 4 [file Image2.TIF]

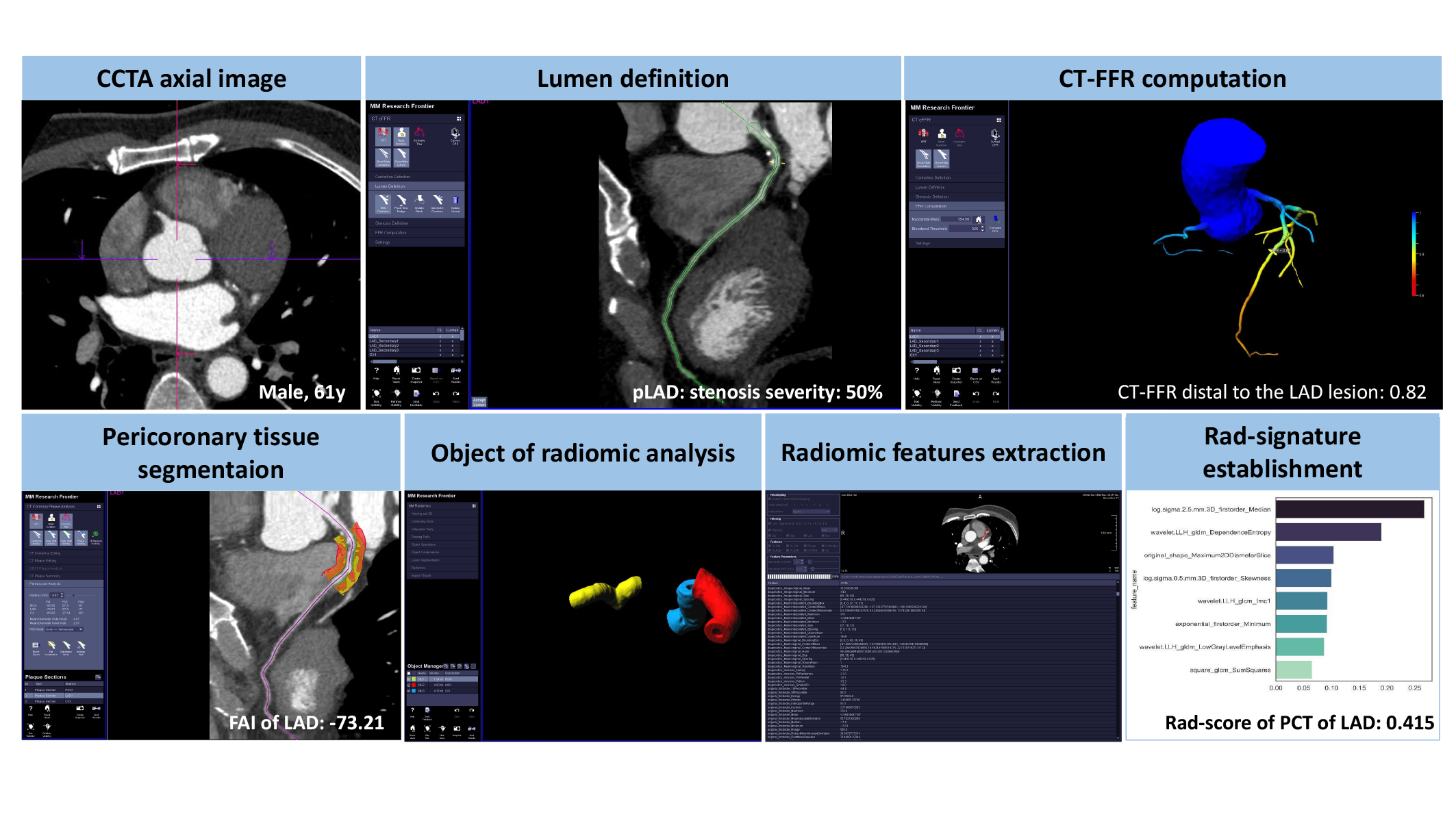

Supplement: Supplementary file 5 [file Image1.TIF]
